# Supplementary material for: Low-temperature nucleation anomaly in silicate glasses shown to be artifact in a 5BaO·8SiO2 glass
Source: Nat Commun. 2021 Apr 1;12:2026. doi: 10.1038/s41467-021-22161-9 (PMC8016887; doi:10.1038/s41467-021-22161-9)
Supplement: Supplementary file 1 — Supplementary Information [file 41467_2021_22161_MOESM1_ESM.pdf]

Supplementary Information

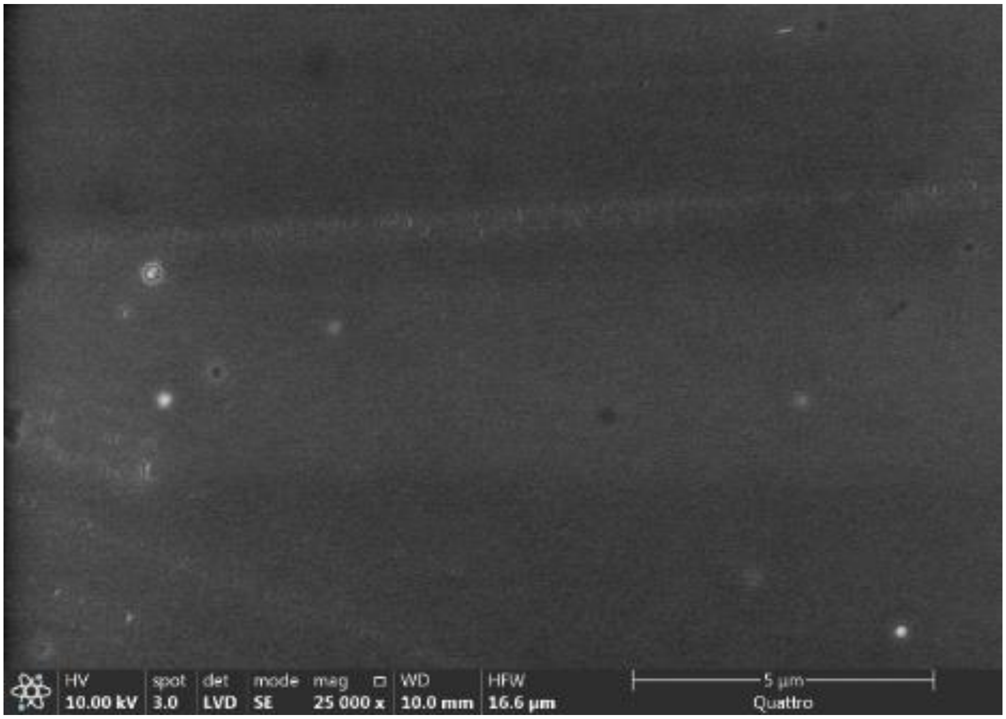

Supplementary Figure 1 Typical SEM image obtained for a $5BaO{\cdot}8SiO_2$ glass nucleated at 948 K for 15 days, and then growth treated at 1073 K.

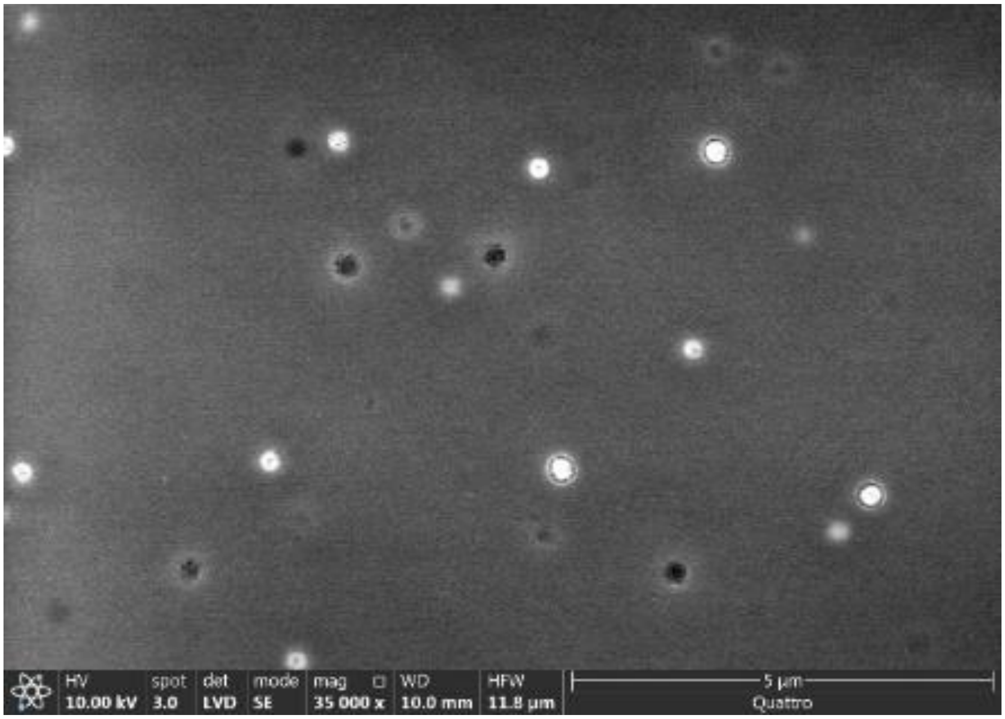

Supplementary Figure 2 Typical SEM image obtained for a $5BaO{\cdot}8SiO_2$ glass nucleated at 948 K for 35 days, and then growth treated at 1073 K.

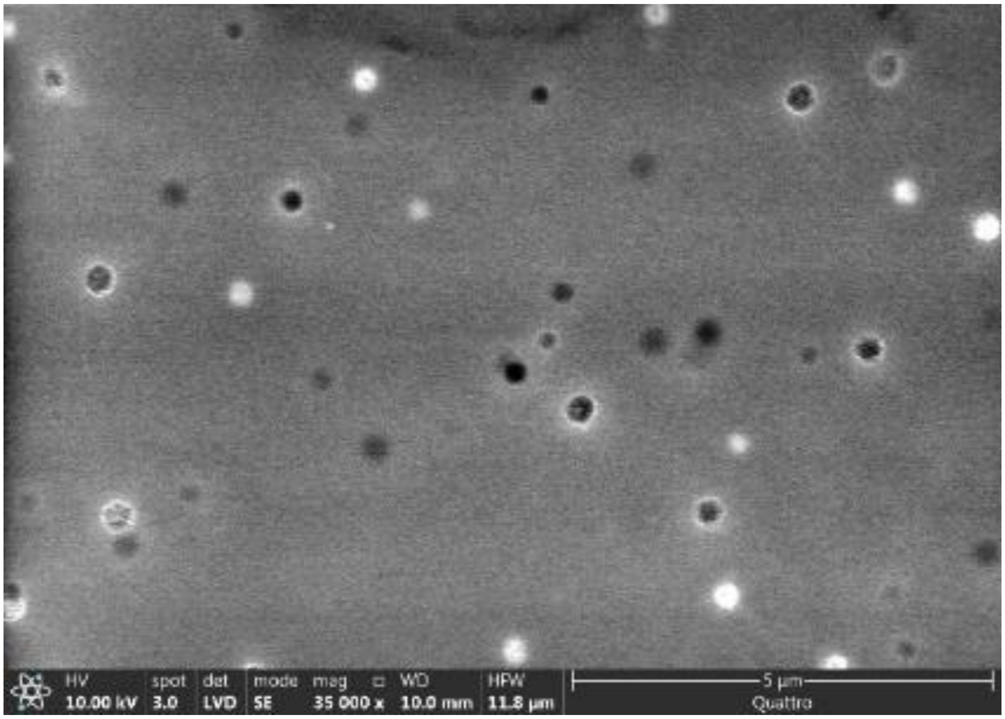

Supplementary Figure 3 Typical SEM image obtained for a $5BaO{\cdot}8SiO_2$ glass nucleated at 948 K for 55 days, and then growth treated at 1073 K.

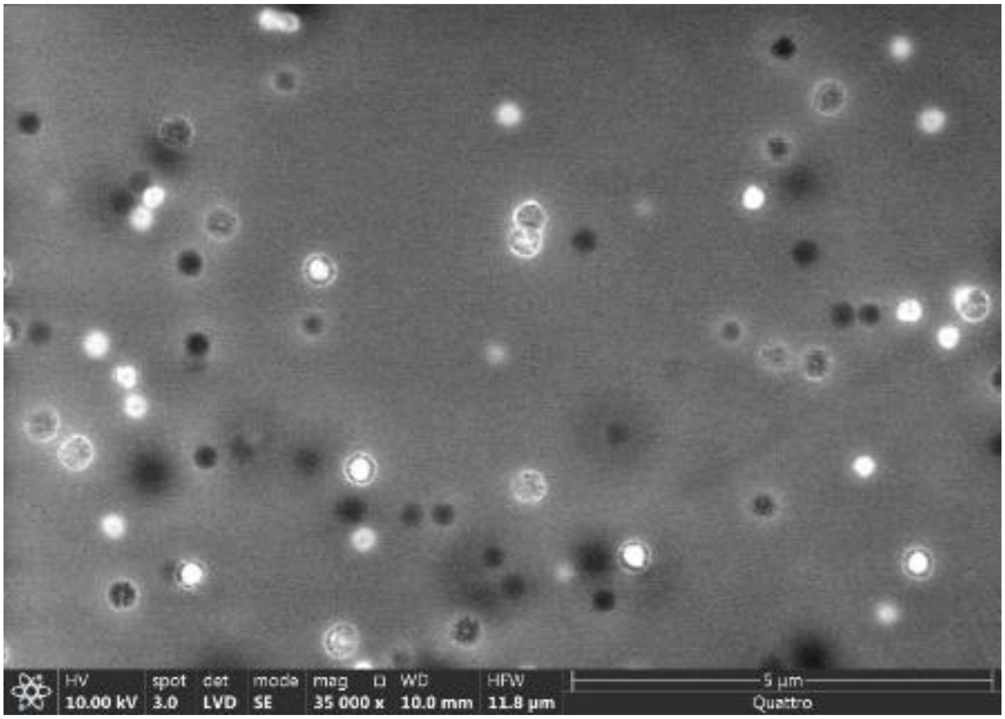

Supplementary Figure 4 Typical SEM image obtained for a $5BaO{\cdot}8SiO_2$ glass nucleated at 948 K for 75 days, and then growth treated at 1073 K.

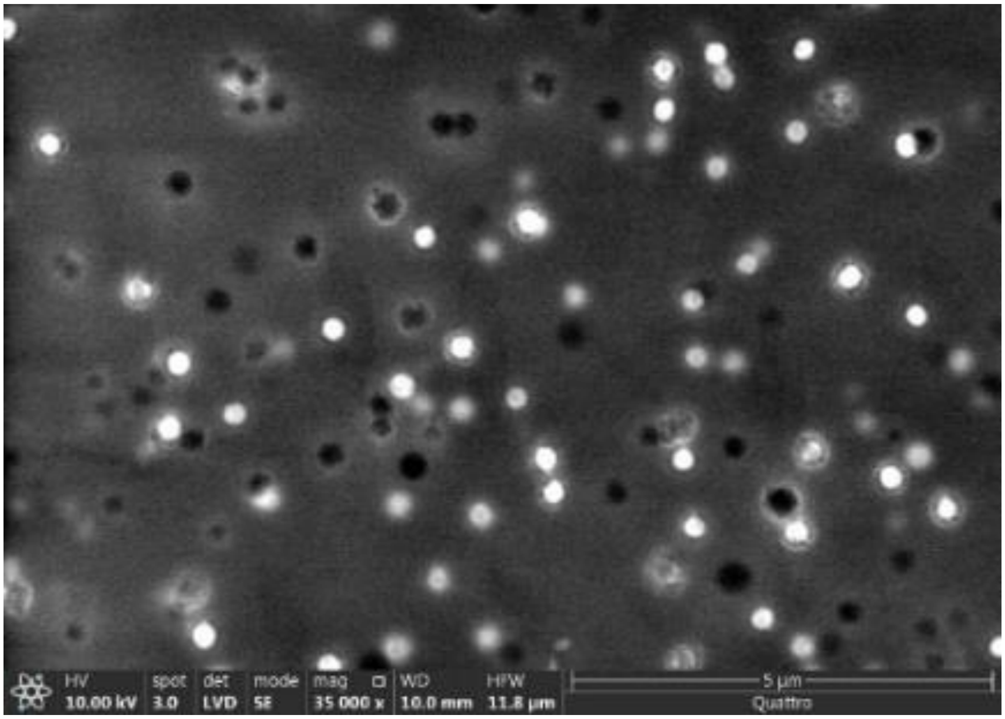

Supplementary Figure 5 Typical SEM image obtained for a $5BaO{\cdot}8SiO_2$ glass nucleated at 948 K for 95 days, and then growth treated at 1073 K.

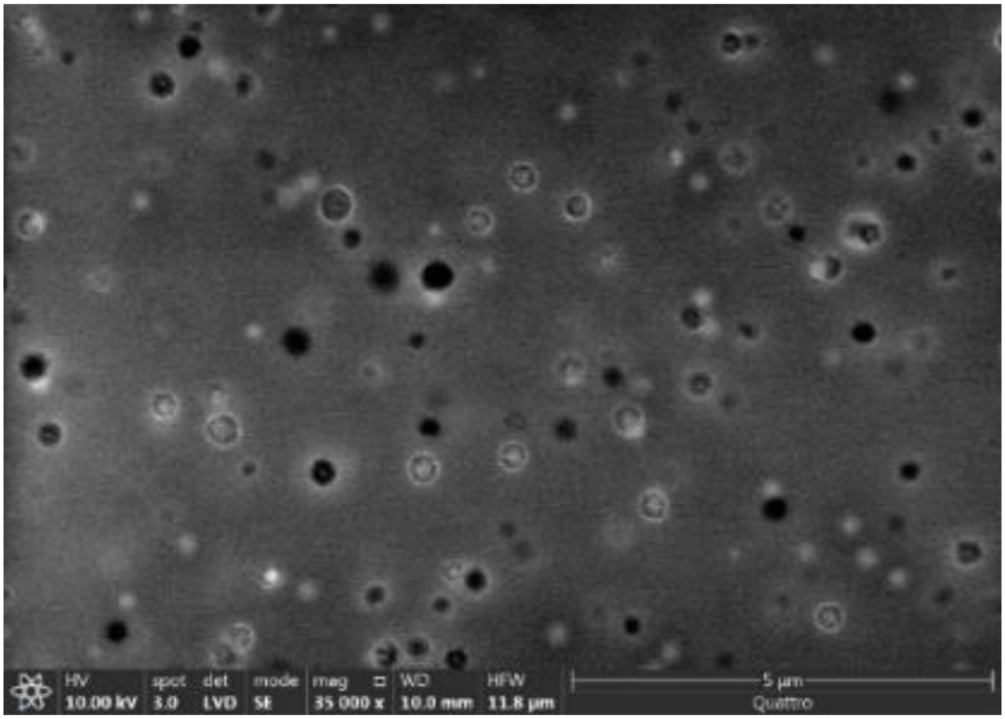

Supplementary Figure 6 Typical SEM image obtained for a $5BaO{\cdot}8SiO_2$ glass nucleated at 948 K for 115 days, and then growth treated at 1073 K.

Supplementary Method 1: the analysis method for the interfacial free energy σ, critical work of cluster formation W^*, and corrected induction time $\theta_{n*(T_N)}$

The data analysis used supplementary eq. 1, which was derived from the Classical Nucleation Theory (CNT) (see Xia *et al.*[1]) .

$$\ln\left(I^{\mathrm{st}}\theta_{n^*(T_G)}\right) = \ln\left\{\left(\frac{\sigma_{T_G}}{\sigma_{T_N}}\right) \div \left(\frac{|\Delta g_v|_{T_G}}{|\Delta g_v|_{T_N}}\right) + \ln\left[\left(\frac{\sigma_{T_G}}{\sigma_{T_N}}\right) \div \left(\frac{|\Delta g_v|_{T_G}}{|\Delta g_v|_{T_N}}\right) - 1\right] + \ln\left[\frac{32\pi}{k_{\mathrm{B}}}\right] + \ln\left[\frac{\left(\sigma_{T_N}\right)^3}{T_{\mathrm{N}}\left(|\Delta g_v|_{T_N}\right)^2}\right] + \zeta_{\mathrm{E}} - 2\right\}$$
$$+ \ln\left[\frac{32}{\pi^2\bar{v}^2}\right] + \frac{1}{2}\ln\left(\frac{\left(\sigma_{T_N}\right)^3 k_{\mathrm{B}}T_{\mathrm{N}}}{\left(|\Delta g_v|_{T_N}\right)^4}\right) - \left(\frac{16\pi}{3k_{\mathrm{B}}}\frac{1}{T_{\mathrm{N}}\left(|\Delta g_v|_{T_N}\right)^2}\right)\left(\sigma_{T_N}\right)^3 \quad . \tag{1}$$

Here, I^{st} is the measured steady-state nucleation rate, $\theta_{n*(T_G)}$ is the measured induction time, σ_{T_N} is the interfacial free energy at the nucleation temperature, σ_{T_G} is the interfacial free energy at the growth temperature, $|\Delta g_v|_{T_N}$ and $|\Delta g_v|_{T_G}$ are the Gibbs driving free energies per unit volume at the nucleation and growth temperature, respectively, k_{B} is Boltzmann's constant, T_{N} is the nucleation temperature, ζ_{E} is Euler's constant (0.5772…), and $\bar{v}$ is the monomer volume.[1] Since σ_{T_N} and σ_{T_G} must be known, the new data presented here at 948 K were analyzed using our previous measurements of I^{st} and $\theta_{n*(T_G)}$ data for temperatures at and above that of the maximum steady-state nucleation rate.[1] Those high temperature data are free from the problems associated with the low temperature data.

The analysis steps were:

(a) Linearly extrapolate the interfacial free energies at 998 K, 1011 K, and 1023 K calculated in Xia *et al.*[1] to obtain an initial estimate for the interfacial free energy at the growth temperature, σ_{T_G} (1073 K for this study).

(b) Use this value of σ_{T_G} and supplementary eq. 1 to calculate the initial value of interfacial free energy at the nucleation temperature, σ_{T_N} (948 K in this study)

(c) Linearly extrapolate the initial value of σ_{T_N} at 948 K (this study) and σ_{T_N} at 998 K, 1011 K, and 1023 K (Xia *et al.*[1]) to a new estimate of σ_{T_G} at 1073 K and 1119 K(the growth temperature used in the earlier study, Xia *et al.*[1])

(d) If the difference between the new value of σ_{T_G} and the previous value of σ_{T_G} at either 1073 K and 1119 K is larger than 10^{-5} J/m^2, use the new set of values for σ_{T_G} at 1073 K and 1119 K , recalculate σ_{T_N} at 948 K , 998 K, 1011 K, and 1023 K using supplementary eq. 1, linearly extrapolate σ_{T_N} to obtain another set of σ_{T_G} . Continue this step until convergence is reached.

After convergence, the values of σ_{T_N} at 948 K, 998 K, 1011 K, and 1023 K, and σ_{T_G} at 1073 K and 1119 K were determined. The critical work of cluster formation, W^*, was calculated using supplementary eq. 2[2], and the interfacial free energy, σ , the Gibbs driving free energy per volume, $|\Delta g_v|$ for each temperature.

$$W^* = \frac{16\pi}{3} \frac{\sigma^3}{|\Delta g_v|^2} . \tag{2}$$

The measured induction time, $\theta_{n^*(T_G)}$, was for the critical size at the growth temperature. To compare with the predictions of CNT, however, the induction time for the critical size at the

nucleation treatment temperature, $\theta_{n^*(T_N)}$, is required. Following Xia *et al.*[1], $\theta_{n^*(T_N)}$ can be calculated from $\theta_{n^*(T_G)}$ using the following expression[2,3]

$$\frac{\theta_{n^*(T_G)}}{\theta_{n^*(T_N)}} = \frac{6}{\pi^2}\left[\xi + \ln\xi + \ln\left(\frac{6W^*}{k_\mathrm{B}T}\right) + \zeta_\mathrm{E} - 1\right], \tag{3}$$

where

$$\xi = \left(\frac{\sigma_{T_G}}{\sigma_{T_N}}\right) \div \left(\frac{\left|\Delta g_v\right|_{T_G}}{\left|\Delta g_v\right|_{T_N}}\right) - 1\ . \tag{4}$$

Supplementary Method 2: Using the Kashchiev expression to calculate the diffusion coefficient from the induction time for the critical size at the nucleation temperature

For spherical clusters, the critical cluster size n^* is given by

$$n^* = \frac{32\pi}{3\bar{v}} \frac{\sigma^3}{\left|\Delta g_v\right|^3} . \tag{5}$$

The forward reaction rate at the critical size, $k_{n^*}^+$, is given by

$$k_{n^*}^+ = \frac{6D}{\lambda^2} O_{n^*} \exp\left(-\frac{W_{n^*+1} - W_{n^*}}{2k_\mathrm{B}T} \right), \tag{6}$$

where D is the diffusion coefficient, λ is the jump distance, O_n is the number of attachment sites (equal to $4n^{2/3}$ for a spherical cluster containing n monomers), and W_n is the work required to form a cluster of size n. From this we can express the diffusion coefficient as a function of the forward reaction rate at the critical cluster size. The Kashchiev[4] expression gives the induction time at the critical size for the nucleating temperature, $\theta_{n^*(T_N)}$, as a function of the transient time, τ_K, or the forward reaction rate at the critical size,

$$\theta_{n^*(T_N)} = \frac{\pi^2 \tau_\mathrm{K}}{6} = \frac{4k_\mathrm{B}Tn^*}{k_{n^*}^+ \bar{v} \left|\Delta g_v\right|} . \tag{7}$$

Relating supplementary equation 6 and supplementary equation 7 gives the diffusion coefficient as a function of the induction time for the critical size at the nucleation temperature,

$$D = \frac{k_\mathrm{B}T\lambda^2 \left(n^*\right)^{1/3}}{6\theta_{n^*(T_N)} \bar{v} \left|\Delta g_v\right|} \exp\left(\frac{W_{n^*+1} - W_{n^*}}{2k_\mathrm{B}T} \right), \tag{8}$$

where the exponential is a number close to one.

Supplementary References

1. Xia, X. *et al.* Time-dependent nucleation rate measurements in $BaO·2SiO_2$ and $5BaO·8SiO_2$ glasses. *J. Non. Cryst. Solids* **525**, 119575 (2019).
2. Kelton, K. F. & Greer, A. L. *Nucleation in condensed matter: applications in materials and biology*, Elsevier, Amsterdam (2010).
3. Shneidman, V. A. & Weinberg, M. C. Induction time in transient nucleation theory. *J. Chem. Phys.* **97**, 3621–3628 (1992).
4. Kashchiev, D. Solution of the non-steady state problem in nucleation kinetics. *Surf. Sci.* 14, 209–220 (1969).
